# Assessment of Ultrasound / Radio-guided Occult Lesion Localization in Non-palpable Breast Lesions

**DOI:** 10.22038/aojnmb.2017.9898

**Published:** 2018

**Authors:** Seyed Ali Alamdaran, Donia Farokh, Ayda Sharifi Haddad, Navid Daghighi, Elaheh Modoodi, Ramin Sadeghi, Mohammad Naser Forghani, Asieh Sadat Fattahi

**Affiliations:** 1 Surgical Oncology Research Center, Mashhad University of Medical Sciences, Mashhad, Iran; 2 Department of Radiology, Faculty of Medicine, Mashhad University of Medical Sciences, Mashhad, Iran; 3 Nuclear Medicine Research Center, Mashhad University of Medical Sciences, Mashhad, Iran; 4 Endoscopic and Minimally Invasive Surgery Research Center, Mashhad University of Medical Sciences, Mashhad, Iran

**Keywords:** Non-palpable breast lesion localization, Radiopharmaceuticals, ROLL, Ultrasound

## Abstract

**Objective(s):**

Controversy exists about the localization of non-palpable breast lesions. In many countries, the gold standard for the diagnosis of these lesions is needle localization due to its accuracy. This study sought to compare the ultrasound- and radio-guided occult lesion localization (ROLL) as a simple method with the conventional procedures in terms of their diagnostic power.

**Methods:**

This study was conducted on 94 patients with non-palpable breast lesions detected by ultrasonography and localized by the combination of ultrasonography and using radiopharmaceuticals. One to ten hours prior to surgery, 0.1-0.2 ml (equivalent to 0.5-1 mCi) of Tc-99m-phytate was injected to the lesion under the guidance of ultrasonography. Then, the lesion was localized using a hand-held gamma probe, and excision of the lesion was performed according to its radioactivity signal. Data analysis was performed using SPSS, version 16.

**Results:**

Benign and malignant pathologic results were observed in 77 (81.9%) and 17(18.1%) of the patients, respectively, and the mean volume of the excised tissue was 26.29±27 mm³. 79 patients had a solitary lesion (84%), 55 in the left breast (58.5%) and 39 in superolateral quadrant (41.5%). The mean size of the lesions was 15.7 mm in diameter (ranging from 4 to 34 mm). Additionally, there was a need to secondary surgery in 3 (3.2%) patients and inappropriate localization in 6 (6.4%) patients (subcutaneous or intra-ductal spread of radiodrug).

**Conclusion:**

Combination of ultrasound- and radio-guided localization methods for localizing non-palpable breast lesions is a simple and acceptable method for localization with no significant complications. For radio-drug spread and subsequent excessive excised tissue volume, subcutaneous and intra-ductal lesions are not suitable indication for ROLL.

## Introduction

The detection of non-palpable breast lesions is a matter of routine due to the novel breast imaging modalities. Accurate localization of these lesions results in the early detection of malignancies, avoidance of unnecessary surgical resection, improvement of free margin rate, patient comfort, and decreased duration of surgery (1). Several methods have been utilized for the localization of occult breast lesions since the past 50 years. The first one (pioneered in 1966) was the implantation of a bent wire through a needle under fluoroscopic guidance (2).

Other techniques, such as Charcot or dye injection, radiological and clinical measurements, wire-guided localization, and radioactive seed localization, are used for localizing lesions (3-5). Most methods have some inaccuracies in the localization of occult breast lesions and are associated with major complications (6).

The placement of wire under the guidance of ultrasonography has some disadvantages for both patients and surgeons including intrusion into the surgical field, needle stick accidents, wire displacement, pneumothorax, the expensiveness of wire, and being an unpleasant experience for patients (7). On the other hand, the accuracy of localization and integral resection with high free margin rate is not satisfactory.

Radio-guided occult lesion localization (ROLL) is a relatively new method, which was first explained by Luini et al. at the European Institute of Oncology in 1998, Milan, Italy (8, 9). In this method, liquid radiopharmaceuticals such as Tc-99m-labeled colloid particles of human serum albumin are injected into the lesion under the guidance of ultrasonography (10, 11). Additionally, this radiopharmaceutical is applied to localize sentinel lymph nodes prior to sentinel node biopsy (SNB) (12). The radioactivity of the radiopharmaceutical is detected by a hand-held gamma probe during surgery, and surgeon can localize the targeted nonpalpable mass and excise it and in the case of malignancy can find the sentinel lymph node in the axilla at the same surgery. (6, 13, 14). In comparison to the wire replacement method, this modality is more cost-effective and is not associated with major complications. This study sought to evaluate the effect of ultrasound- and radio-guided localization on accurate localization of non-palpable breast lesions.

## Methods

This study was conducted on 94 patients with non-palpable breast lesions detected by mammography or ultrasonography with the indication for surgical excision (unfavorable pathology report of core needle biopsy, intraductal or intracystic lesion, patient request) according to the Breast Imaging Reporting and Data System (BI-RADS) lexicon 2012. This study was approved by ethics committee of Mashhad University of Medical Science.

In all the patients, the characteristics of the lesions were assessed by Mind ray DC7 or Class C ESTE My Lab ultrasound systems (10 or 12 MHz probe) at Omid Hospital affiliated to Mashhad University of Medical Sciences, Mashhad, Iran.

The procedures of localization and resection were performed by an interventional radiologist and two breast surgeons in a breast clinic. Tc-99m-labeled sodium phytate was used for the interventional localization of the lesion. A single dose of 0.1-0.2 ml (equivalent to 0.5-1 mCi) of the radiopharmaceutical was injected into the center of the lesion. This procedure was performed by using an insulin syringe and a 23 gauge needle under the guidance of ultrasonography considering the size of the lesion and the interval between localization and resection.

Moreover, 0.1 ml of air was injected in combination with the radiopharmaceutical to assure accurate localization, for desirable distribution of radiopharmaceutical, and appropriate radiopharmaceutical injection. Radiopharmaceutical injection was performed 1-10 hours before the surgery, and the duration of localization was documented for each patient. The extent of incision was determined according to lesion location, which was reported by imaging prior to the surgery, and the location of obvious radioactivity inside the body during surgery.

The surgeon used a hand-held gamma probe during the excision to detect the area of maximum radioactivity, known as hotspot. The typical incision location is the peri-areolar region, especially for non-malignant lesions. The gamma probe is used repeatedly during the surgery to determine the location of hotspot. The extent of the lesion is determined by frequency and intensity of radiation detected by gamma probe.

After the excision and before suturing the wound, resection bed was explored by gamma probe for any remnant radioactivity. If any radiation was detected, the surgical resection would be repeated until no radioactivity was detected; therefore, the wound was closed after complete excision. The duration of surgery and secondary excision, if needed, was documented for each patient. We routinely used frozen section biopsy in all of known oncosurgery cases. The samples were sent to a pathologic laboratory for the investigation of pathologic results. The obtained data from ultrasonography, mammography, surgery, and pathology were analyzed by SPSS, version 16.

## Results

The mean age of the patients was 45.9 years (ranging from 21 to 70 years). The most common presentation of the lesion was pain 49 (52.1%). Most of the patients had a solitary lesion 79 (84%), mostly in the left breast 55 (58.5%) and superolateral quadrant 39 (41.5%). The mean size of the lesions was 15.7 mm in diameter (ranging from 4 to 34 mm).

The pathologic results demonstrated that the lesions were malignant in 17 (18.1)% of the patients. 14 were invasive intraductal carcinoma and 3 were in situ ductal carcinoma. In this study, the patients did not reveal margin involvement after the excision except in 2 (11.8) of malignant patients. Both of these patients had benign pathology on core needle biopsy but found to have in situ breast cancer in surgical specimen. Secondary surgery was performed in this two patients because margin involvement was noted in pathology report. In addition, inappropriate localization occurred in another six patients due to the leakage of radiotracer into the subcutaneous tissue in two and the injection of radiotracer into the breast lesion and adjacent mammary ducts in four patients. Therefore, the surgeon was unable to detect the lesion margin accurately. Accordingly, in these six patients, larger volumes of breast tissue were resected. Secondary surgery was performed in one of six patients because the target lesion (Sclerosing Adenosis) was not excised in primary surgery. Overall, improper localization were occurred in 6 (6.4%) patients, and re-excision were done in 3 (3.2%) patients. As we routinely used frozen section biopsy in all of in known oncosurgery cases, margin involvement and re -excision surgery were not occurred in anyone of known malignant patients.

The mean durations of localization and operation were 1.80±0.42 min (ranging from 1 to 2.5 min) and 26.78±8.10 min (ranging from 10 to 45 min), respectively. Furthermore, the mean volume of excised tissue was 26.29±27 cm³ in all the patients. We had no other complications during procedure related to radio-isotope injection.

## Discussion

Currently, occult breast lesions, which are suspicious for malignancy and have the indication for biopsy or excisional surgery, are revealed by routine ultrasonography and mammography screening programs for early detection of malignant non-palpable breast lesions (1). The accurate localization of these lesions is essential for complete resection; therefore, new diagnostic methods have been developed.

Various modalities are used for lesion localization, all of which are accompanied with some advantages and disadvantages. The most common method is wire-guided localization, which suffers from several limitations including difficult insertion, inaccurate wire replacement, and wire migration and displacement. Additionally, patients usually find the procedure uncomfortable and the wire may harm a member of the surgical team or pathologist during the examination of the obtained specimen (needle stick). In this method, margin involvement may be extended and the risk of recurrence after secondary surgery is relatively high (10, 11). The localization of non-palpable breast lesions by the ultrasonography-guided injection of radiotracer into the lesion is a simple method with more merits and less complications in comparison to the wire-guided localization method. The localization of non-palpable breast lesions by the combination of ultrasonography- and radio-guided methods was reported to be accurate in 95-99% of cases (13, 15).

Moreover, the incidence of margin involvement after excision and secondary surgery is decreased by using this method (7, 11, 16, 17). Additionally, This procedure leads to reduced pain and discomfort, improved cosmetic results, improved detection of sentinel lymph nodes at the same primary procedure for lesion localization (14, 18-22) decreased duration of localization (7, 13, 14, 16, 17, 23, 24), facilitated tumor localization and surgical techniques, reduced costs, and decreased duration of surgery, and hospitalization (6, 25).

Regarding the literature, the incidence rate of margin involvement after resection was 0-25% (13, 14, 20, 21, 26). In our study, we only had margin involvement in 2 malignant patients (11.8%). these had benign lesions in core needle biopsy but found to be malignant after complete evaluation of resected specimen. We routinely used frozen section biopsy in oncosurgery cases, that might be the reason behind our low incidence of margin involvement and re -excision surgery in known malignant patients.

Nevertheless, this method cannot completely replace wire-guided localization due to several disadvantages, such as failure to discriminate between extensive breast lesions, micro-calcifications, and huge breasts. Furthermore, bias in estimating the depth of lesion and radiopharmaceutical extravasation into the adjacent breast quadrants under the guide of stereotaxic due to compression on breast tissue (17, 22), short half-life of the radiopharmaceutical leading to the necessity of excision performance in few hours after the injection, and invisibility of the radiopharmaceutical on mammograms are the other drawbacks of this procedure (6).

In this study, the incidence of improper localization (6.4%) was higher than that reported by Zagajnar (3.9%), Franco (6%), Rumpul (4.1%), Martinez, Moreno, Gallegos, Ronka, Thind, and Strnad (7, 13, 16, 17, 23, 26-29). The rate of secondary surgery in the present study (3.2) was lower compared to that revealed by Franco (11.1%), Martinez (7.6%), Gray (9.6%), Hughes (7.8%)(7, 16, 30, 31).

In this study, the mean excised tissue volume (26.29±27 cm³) was higher than those reported by others (16, 17, 23). It is probably due to patient selection bias as in subcutaneous and intraductal lesions with inappropriate radio-drug spread, limiting the exact mass was difficult, so the surgeon had to excise larger volume of the breast tissue that showed radioactivity. We don’t recommend radio-guided localization in suspicious intra-ductal and subcutaneous lesions (32), due to possible leakage and larger volume excision.

## Limitations of the Study

This study was not a multicentric investigation; furthermore, the smaller size of molecules containing radiopharmaceutical resulted in leakage of radiopharmaceutical in breast tissue, in some patients.

## Conclusion

Ultrasound/Radio-guided localization of non-palpable breast lesions is an inexpensive and simple method with acceptable results. Subcutaneous and intra-ductal lesions may not be suitable indication for ROLL.
